# ELF3 (E74 like ETS transcription factor 3) promotes tumor growth and autophagy in pancreatic cancer

**DOI:** 10.1186/s13046-026-03806-3

**Published:** 2026-08-18

**Authors:** Yuru Shang, Pingping Zhang, Yanhui Yang, Yibao Fan, Zhichao Yang, Hongshi Sun, Jia Wang, Zhengming Yu, Jiale Wu, Wenxu Wang, Ulf Dietrich Kahlert, Gautam Sethi, Michael Linnebacher, Zhe-Sheng Chen, Daniel J Klionsky, Xianbin Zhang

**Affiliations:** 1 Department of Gastrointestinal Surgery, South China Hospital, Medical School, Shenzhen University, Institute of Pharmacy, Shandong University of Traditional Chinese Medicine, Jinan, China; 2 Guangdong Key Laboratory for Biomedical Measurements and Ultrasound Imaging, School of Biomedical Engineering, National-Regional Key Technology Engineering Laboratory for Medical Ultrasound, Shenzhen University Medical School, Shenzhen, China; 3 Department of Gastroenterology, Changhai Hospital, Naval Medical University, Shanghai, China; 4 Department of Trauma Surgery, The First Affiliated Hospital, College of Clinical Medicine, Henan University of Science and Technology, Luoyang, China; 5 Department of General Surgery, Molecular Oncology and Immunotherapy, Rostock University Medical Center, Rostock, Germany; 6 Molecular and Experimental Surgery, Clinic for General-, Visceral-, Vascular-, and Transplant-Surgery, Medical Faculty, University Hospital Magdeburg, Research Campus STIMULATE, University of Magdeburg, Magdeburg, Germany; 7 Department of Pharmacology and NUS Centre for Cancer Research, Yong Loo Lin School of Medicine, National University of Singapore, Singapore, Singapore; 8 Department of Pharmaceutical Sciences, College of Pharmacy and Health Sciences, St. John’s University, Queens, USA; 9 Life Sciences Institute and Department of Molecular, Cellular and Developmental Biology, University of Michigan, Ann Arbor, USA; 10 Traditional Medicine Translational Research Center, Institute of Pharmacy, Shandong University of Traditional Chinese Medicine, Jinan, China; 11 Center for Medical Artificial Intelligence, Shandong University of Traditional Chinese Medicine, Qingdao Academy of Chinese Medical Sciences, Shandong University of Traditional Chinese Medicine, Qingdao Key Laboratory of Artificial Intelligence Technology for Chinese Medicine, Qingdao, China; 12 College of Chinese Materia Medica, Xinjiang University of Traditional Chinese Medicine, Kashi, China

## Abstract

**Background:**

Despite the research progress of the past decades, the prognosis for pancreatic ductal adenocarcinoma remains poor. Identifying novel targets and exploring the underlying mechanisms is therefore of great importance. ELF3 (E74 like ETS transcription factor 3) is a member of the E-twenty-six (ETS) family and previous studies suggest that ELF3 may play a promoting role in malignant epithelial tumor; however, the effect and exact mechanism have not been determined in pancreatic cancer.

**Methods:**

ELF3 expression and its prognostic relevance in pancreatic cancer were analyzed using clinical datasets. The role of ELF3 in autophagy regulation was evaluated using molecular and cellular approaches in vitro and in vivo. Computer-aided drug design was performed to identify potential ELF3 inhibitors, whose anti-tumor efficacy was subsequently evaluated.

**Results:**

In the present study, it is observed that high expression of ELF3 leads to a poor overall survival in pancreatic cancer patients. Functional experiments demonstrated that knockdown of ELF3 blocks autophagy. In addition, SRT2183 was identified as a potential ELF3 inhibitor using molecular docking. In vitro and in vivo results showed that SRT2183 blocks autophagy and exerts anti-carcinoma effect.

**Conclusions:**

These findings demonstrate that ELF3 promotes autophagy through transcriptional regulation of ATG10 and identify ELF3 as a potential therapeutic target in pancreatic cancer. SRT2183, an ELF3-targeting compound that suppresses autophagic flux and tumor growth, represents a promising candidate for further therapeutic development.

**Supplementary Information:**

The online version contains supplementary material available at https://doi.org/10.1186/s13046-026-03806-3.

## Introduction

Pancreatic ductal adenocarcinoma (PDAC) is among the most frequent causes of cancer-related death with an increasing global health burden [1, 2]. Chemotherapeutic drugs, including gemcitabine (GEM) and 5-fluorouracil (5-FU), remain the cornerstone of treatment for PDAC patients [3]. However, a large subset of cancer patients is insensitive to these drugs and results in poor clinical outcomes [4–6]. One of the main reasons is the biological complexity of PDAC and the incomplete understanding of its regulatory mechanisms which require further investigation. Therefore, it is critical to elucidate the underlying mechanisms of PDAC and explore novel therapeutic targets.

Autophagy is an evolutionarily conserved cellular degradation pathway orchestrated by autophagy-related (ATG) proteins, which regulate phagophore formation and expansion to sequester selected cargo into autophagosomes for subsequent lysosomal degradation [7–9]. Depending on the type and stage of cancer, autophagy can either suppress or promote tumor progression [10, 11]. In PDAC, autophagy is increasingly recognized as a survival mechanism that enables tumor cells to cope with metabolic stress and sustain growth under adverse conditions [12, 13]. However, clinically applicable autophagy inhibitors remain unavailable to date. Elucidating the molecular mechanisms regulating autophagy in PDAC will not only advance our theoretical understanding of tumorigenesis but, more importantly, provide crucial molecular targets for the development of novel therapies.

ELF3 (E74 like ETS transcription factor 3), a member of the E-twenty-six (ETS) family of transcription factors, has emerged as a potential therapeutic target for PDAC [14–17]. For example, Dudgeon et al. reported that Netrin-1 promoted liver metastasis of pancreatic cancer by activating the ELF3 signaling pathway in hepatic stellate cells [16]. Yamaguchi et al. observed that 12-O-tetradecanoylphorbol 13-acetate induced CLDN7 (claudin 7) expression—a protein involved in pancreatic cancer cell motility and invasion—through ELF3 activation [17]. These studies suggest that ELF3 contributes to the malignant phenotypes of PDAC cells. Yet, no studies have directly explored whether ELF3 regulates autophagy and tumor growth in PDAC. Additionally, no small-molecule inhibitors targeting ELF3 have been reported. Thus, in the present study, we evaluated if and how ELF3 regulates autophagy and tumor progression in pancreatic cancer. Furthermore, we identified a potential ELF3 inhibitor and elucidated its underlying mechanism of action.

## Methods

### Cell culture and reagents

The human pancreatic cancer cell lines and cell culture conditions were reported in our previous study [18, 19]. To establish cell lines in which ELF3 was knocked down or overexpressed, *ELF3* shRNA plasmids sh*ELF3* #1 (KD#1), sh*ELF3* #2 (KD#2), and CV129 encoding ELF3 (ELF3-OE) were obtained from GeneChem (Shanghai, China). The shRNA sequences are presented in Table S1 and the details of the process are reported in our previous study [20]. The reagents used in this study were purchased from Selleck, MedChemExpress, Sigma Aldrich and Merck Millipore, respectively (Table 1).

**Table 1:** Basic information and clinical status of candidate compounds

Compound	SMILE	PubChem CID	Clinical trials	FDA proved	Company (Catalog No.)
SRT2183	C1CN(CC1O)CC2 = CSC3=NC(= CN23)C4 = CC = CC=C4NC(= O)C5 = CC6 = CC = CC=C6C=C5	24180126	0	Investigational	Selleck (S8270)
SRT2104	CC1 = C(SC(= N1)C2 = CN = CC=C2)C(= O)NC3 = CC = CC=C3C4 = CN5C(= CSC5 = N4)CN6SCCOCC6	25108829	14	Investigational	Selleck (S7792)
SRT3025	COCCCC1 = C(N = C(S1)C2 = CC = CC=C2)C(= O)NC3 = CC = CC=C3C4 = NC5=C(S4)N = CC(= C5)CN6CCCC6	46245047	1	Investigational	Selleck (S8481)
Gemcitabine (GEM)	C1 = CN(C(= O)N=C1N)[C@H]2 C([C@@H]([C@H](O2)CO)O)(F)F	60750	3379	Approved	MedChemExpress (HY-B0003)
5-Fluorouracil (5-FU)	C1 = C(C(= O)NC(= O)N1)F	3385	2592	Approved	Selleck (S1209)
Oxaliplatin (OXA)	C1CC[C@H]([C@@H](C1)[NH-])[NH-].C(= O)(C(= O)O)O.[Pt + 2]	9887053	2991	Approved	Selleck (S1224)
Irinotecan (IR)	CCC1 = C2CN3C(= CC4 = C(C3 = O)COC(= O)[C@@]4(CC)O)C2 = NC5=C1C = C(C = C5)OC(= O)N6CCC(CC6)N7CCCCC7	60838	1985	Approved	Selleck (S5026)
Chloroquine (CQ)	CCN(CC)CCCC(C)NC1 = C2C = CC(= CC2 = NC=C1)Cl	2719	248	Investigational	SigmaAldrich (C6628)
LW6	COC(= O)C1 = CC(= C(C = C1)O)NC(= O)COC2 = CC = C(C=C2)C34CC5CC(C3)CC(C5)C4	16124726	0	Investigational	Merck Millipore (400083)
PPQ-102	CC1 = CC = C(O1)C2C3 = C4C(= C(N3C5 = CC = CC=C5N2)C6 = CC = CC=C6)C(= O)N(C(= O)N4C)C	16016583	0	Investigational	MedChemExpress (HY-14179)
GSK717	CN(CCC1 = NC2 = CC = CC=C2N1CC(= O)NC3 = CC4=C(CCC4)C = C3)C(= O)C5 = CC = CC=C5	102369397	0	Investigational	MedChemExpress (HY-136555)
Aspirin (ASP)	CC(= O)OC1 = CC = CC=C1C(= O)O	2244	2047	Approved	MedChemExpress (HY-14654)
Pyrocatechuic Acid (PCA)	C1 = CC(= C(C(= C1)O)O)C(= O)O	19	0	Investigational	MedChemExpress (HY-Y0202)

### Western blot

Western blot was performed according to our previous publication [20] with the following primary antibodies: anti-ELF3 (Novus Biologicals, Littleton, USA, catalog no. NBP1-30873, dilution: 1000×), anti-survivin (Proteintech, Chicago, USA, catalog no. 10508-1-AP, dilution: 1000×), anti-β-actin (Proteintech, catalog no. 66009-1-Ig, dilution: 20000×), anti-ATG10 (Abcam, Cambridge, UK, catalog no. ab124711, dilution: 1000×), anti-p62 (Proteintech, catalog no. 18420-1-AP, dilution: 5000×), anti-LC3 (Proteintech, catalog no. 14600-1-AP, dilution: 1000×), anti-p27 (Proteintech, catalog no. 25614-1-AP, dilution: 1000×), anti-p53 (Proteintech, catalog no. 10442-1-AP, dilution: 5000×), anti-SIRT1 (Proteintech, catalog no. 13161-1-AP, dilution: 5000×).

### Evaluation of anti-carcinoma efficacy in vitro

PANC-1 or MIA PaCa-2 cells transfected with KD#1, KD#2, or NC were plated in 24-well plates for 48 h, then stained using the BeyoClick™ EdU kit (Beyotime, Shanghai, China, catalog no. C0071S). To evaluate cell viability, 4 × 10³ cells/well were seeded in 96-well plates and treated with distinct compounds. The cell viability was analyzed by measuring absorbance at 450 nm using a Synergy LX multimode reader (BioTek, Winooski, USA).

To evaluate cell apoptosis, 2.5 × 10⁵ MIAPaCa-2 or PDA cells/well were plated in 6-well plates, treated with DMSO (Sham) or SRT2183 for 48 h and stained with Annexin V-APC/PI (Elabscience, Houston, USA, catalog no. E-CK-A217). Flow cytometry was subsequently performed (NovoCyte Agilent, Santa Clara, USA). Apoptosis was also assessed using the JC-1 kit (Beyotime, catalog no. C2003), with fluorescence images captured (Leica Microsystems, Wetzlar, Germany).

### Subcutaneous xenograft and orthotopic xenograft models

All animal procedures were approved by the Institutional Animal Care and Use Committee of Shandong University of Traditional Chinese Medicine (SDUTCM20251230003) and complied with ethics guidelines. BALB/c nude mice (male, 6 weeks old; Vital River, Beijing) were subcutaneously injected with 1 × 10⁶ PANC-1 cells (transfected with KD#1, KD#2, or NC) in 100-µL suspensions. Tumor volume was measured every 5 days. Mice were euthanized on day 40, and tumor weight or volume was assessed to determine ELF3 knockdown effects.

To evaluate the anti-carcinoma efficacy of SRT2183, an orthotopic pancreatic cancer model was established [13]. Four days post-operation, mice received intraperitoneal injections of DMSO (Sham) or distinct concentrations of SRT2183. Treatment-related distress was scored as previously reported [21]. To evaluate the therapeutic effects of SRT2183, a subcutaneous xenograft model was established as described above. When tumors reached 100–200 mm³, mice received intraperitoneal DMSO (Sham) or SRT2183. On day 24 post-injection, blood was collected for evaluating the level of aspartate aminotransferase (ALT) or alanine aminotransferase (AST).

### Immunohistochemistry (IHC) and immunofluorescence (IF) assay

IHC and IF assay were performed as described by Yuan et al. [20]. Briefly, tumor tissue was stained with the primary antibody as follows: anti-ELF3 (Novus Biologicals, catalog no. NBP1-30873, dilution: 400×), anti-p62 (Proteintech, catalog no. 18420-1-AP, dilution: 200×), anti-LC3 (Proteintech, catalog no. 14600-1-AP, dilution: 200×), anti-Ki67 (Proteintech, catalog no. 27309-1-AP, dilution: 200×) and anti-ATG10 (Abcam, catalog no. ab124711, dilution: 400×), followed by a secondary antibody from the dual-label multiplex immunohistochemistry kit (YEPCOME, Shanghai, China catalog no. YP-DB002). The images were obtained with the support of an ECLIPSE Ti2 confocal microscope (Nikon, Tokyo, Japan).

### Evaluation of tandem RFP-GFP-LC3 fluorescence

For evaluation of autophagic flux, 2 × 10^5^ PANC-1 cells or MIA PaCa-2 cells per well transfected with KD#1 or NC were cultured in a 12-well plate. These cells were cultured in medium with 10% FBS or 1% FBS for 48 h. Subsequently, cells were transfected with the help of liposomal transfection reagent (Yeasen, Shanghai, China, catalog no. 40802ES08) and ptfLC3 plasmid (Addgene, Cambridge, UK, catalog no. 21074), which was a gift from Tamotsu Yoshimori [22]. The autophagic flux was observed using an ECLIPSE Ti2 confocal microscope.

### Chromatin immunoprecipitation assay

To examine whether ELF3 regulates *ATG10* transcription, MIA PaCa-2 cells were transfected with ELF3-OE or empty vector and cultured to 50–60% confluence in 15-cm dishes. Cells were treated with DMSO or SRT2183 for 48 h before analysis. Chromatin immunoprecipitation was performed using the ChIP assay kit (Beyotime, catalog no. P2078). Briefly, cells were crosslinked with 1% formaldehyde (20 min, room temperature) and quenched with 125 mM glycine. Chromatin was sheared to 200–600 bp fragments using ultrasonic disruption, with fragmentation efficiency verified by 1.2% agarose gel electrophoresis. For immunoprecipitation, 50–100 µg chromatin was incubated overnight at 4 °C with an anti-ELF3 antibody or a normal IgG control (Abcam, catalog no. ab171870), followed by incubation with Protein A/G Magnetic Beads (Thermo Fisher Scientific, Waltham, USA, catalog no. 26162). After sequential washes with low-salt (150 mM), high-salt (500 mM), LiCl (250 mM) and TE (10 mM Tris-Cl, 1 mM EDTA, pH 8.0) buffers, DNA-protein complexes were eluted and reverse crosslinked using 10% Chelex (Sigma, catalog no. C7901) and proteinase K (Beyotime, catalog no. ST533) treatment. Putative ELF3-binding sites in *ATG10* and *ELF3* promoters were predicted using the EPD database, with site-specific primers designed for PCR analysis of ChIP samples and corresponding input DNA.

### Molecular docking

The structure of ELF3 was predicted using the AlphaFold Protein Structure Database (https://alphafold.ebi.ac.uk/entry/P78545, model: AF-P78545-F1) and the docking pocket was predicted by ProteinsPlus. The 3D structures of the chemical compounds were obtained from the PubChem database (Table 1). Subsequently, molecular docking was conducted to evaluate the binding affinity between ELF3 and distinct chemical compounds with assistance of AutoDock Vina. In general, affinity < − 7 kcal/mol suggests a strong predicted binding between compounds and targets, affinity < − 5 kcal/mol suggests a moderate predicted binding between compounds and targets, and affinity > − 5 kcal/mol suggests no predicted binding between compounds and targets [23].

### Cellular thermal shift assay

To assess the thermal stabilization of ELF3 protein upon SRT2183 binding, MIA PaCa-2 cells were cultured in 10 cm dishes until reaching 50–60% confluence. After 48 h of treatment with vehicle control or SRT2183, cells were harvested and lysed on ice using M-PER buffer (Thermo Fisher Scientific, catalog no. 78501) supplemented with phenylmethylsulfonyl fluoride, protease inhibitor cocktail, and phosphatase inhibitor cocktail. The heat treatment was carried out at incremental temperatures: 37, 40, 45, 50, 55, 60 and 65 °C for 3 min in a metal bath. Following heating, samples were equilibrated at room temperature for 3 min and then centrifuged at 14000×g for 10 min at 4 °C. The resulting supernatant fractions were collected and analyzed by western blot using the specified antibodies as described.

### Statistical analysis

Statistical analyses were conducted with GraphPad Prism (version 10.1) or R software (version 4.3.2). The prognostic significance of ELF3 was evaluated by Kaplan-Meier survival curves with statistical significance determined by log-rank test. To further adjust for potential confounders, a multivariate Cox proportional hazards regression model was constructed. To identify differentially expressed genes, GSE64558, which included two wild-type control samples (NC#1 and NC#2) and three independent *ELF3*-knockout (ELF3-KO#1, ELF3-KO#2 and ELF3-KO#3) clones, were obtained from Gene Expression Omnibus database, and the DESeq2 R package was used. Genes with |log2FC| > 1 and padj value < 0.05 were considered as differentially expressed genes. These genes were presented by lollipop plot and volcano plot, respectively. The Gene Set Enrichment Analysis (GSEA) of genes was performed using the clusterProfiler R package.

For the in vitro and in vivo results, data are presented as medians and 95% confidence intervals (CIs). The statistical significance of the difference between two groups was determined by the Mann-Whitney U test. For comparisons involving more than two groups, a Kruskal-Wallis test was performed, followed by Mann-Whitney U test when appropriate. Statistical significance was defined as a *P*-value less than 0.05 after applying Bonferroni correction for multiple comparisons (α = 0.05/n, where n represents the number of meaningful comparisons).

## Results

### ELF3 is a promising target for the treatment of PDAC

**Fig. 1:**
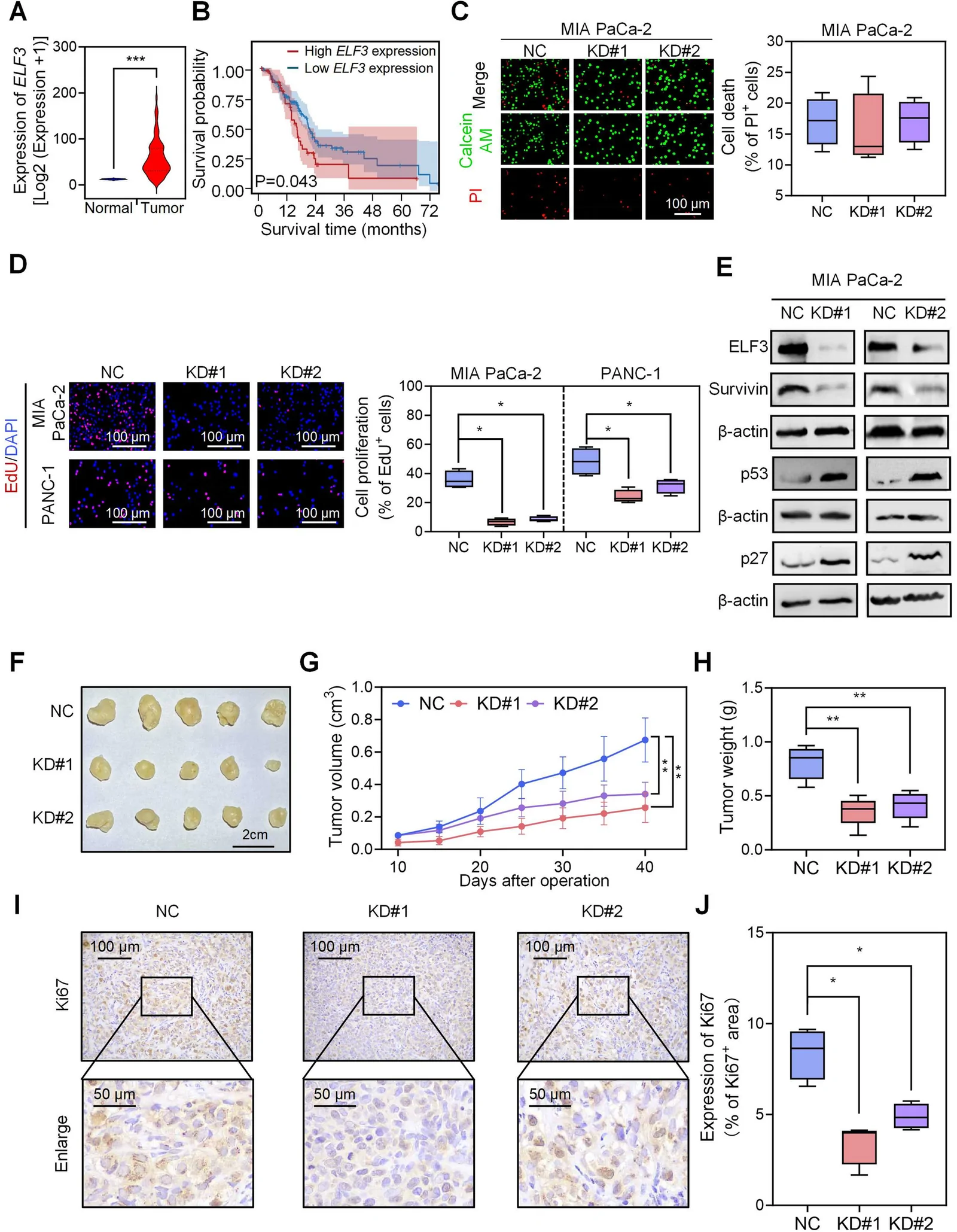
ELF3 is an oncogene that promotes the growth of pancreatic tumor. **A** The Student’s t-test demonstrated that ELF3 was significantly upregulated in PDAC tissues. **B** Kaplan-Meier and log-rank test indicated that high *ELF3* expression was significantly associated with poor prognosis in PDAC patients. **C** Calcein AM/PI staining was used to assess cell viability that KD-ELF3 did not significantly induce apoptosis. **D** EdU staining indicated that KD-ELF3 blocked cell proliferation. **E** Western blot analysis showed that KD-ELF3 decreased the expression of survivin, however, it induced the level of p53 and p27 in MIA PaCa-2. **F** and **G** KD-ELF3 significantly reduced tumor volume and (**H**) tumor weight. **I** and **J** IHC analysis proved that KD-ELF3 significantly inhibited tumor proliferation. *N* = 4 for (**C**, **D**);*N* = 5 for (**G**, **H**, **J**). *, *P* < 0.05; **, *P* < 0.01; ***, *P* < 0.0001

In order to evaluate the role of *ELF3* in PDAC, we obtained 160 PDAC tissues and 362 normal tissues from The Cancer Genome Atlas (TCGA) and The Genotype-Tissue Expression (GTEx) database, respectively (Fig. S1A). We observed significantly higher *ELF3* expressions in PDAC tissues than in normal tissues (Fig. 1A). Kaplan-Meier analysis revealed that PDAC patients with high *ELF3* expression experienced significantly worse overall survival than those with low expression (Fig. 1B). This finding is supported by the data from the Gene Expression Omnibus (GEO) database (GSE85916; Fig. S1B). Additionally, to adjust for potential confounding factors affecting patient survival, we performed Cox regression and observed that *ELF3* is the independent risk factor affecting patient prognosis (Fig. S1C). Collectively, these findings establish ELF3 as a potential therapeutic target in PDAC.

To validate these observations, *ELF3* was genetically silenced in carcinoma cells using two independent short hairpin RNAs (KD#1 and KD#2), followed by assessment of apoptosis and proliferation. Notably, knockdown of ELF3 (KD-ELF3) did not significantly increase apoptosis (Fig. 1C). In contrast, KD-ELF3 significantly inhibited cell proliferation (Fig. 1D) and altered the expression of cell cycle regulatory proteins, such as p53 and p27 (Fig. 1E).

To investigate the function of ELF3 in vivo, we established xenograft mouse models by subcutaneously injecting either negative control (NC) or ELF3-knockdown cells. Consistent with our in vitro findings, KD-ELF3 significantly reduced tumor volume (Fig. 1F and G) and weight (Fig. 1H). Furthermore, IHC analysis demonstrated that KD-ELF3 significantly decreased Ki67 expression (Fig. 1I and J) which indicated inhibition of proliferative activity in the tumors. Together, our findings demonstrate that KD-ELF3 significantly inhibited tumor growth and that ELF3 is a promising therapeutic target for PDAC.

### ELF3 promotes autophagy and enhances the transcription of ATG10

**Fig. 2:**
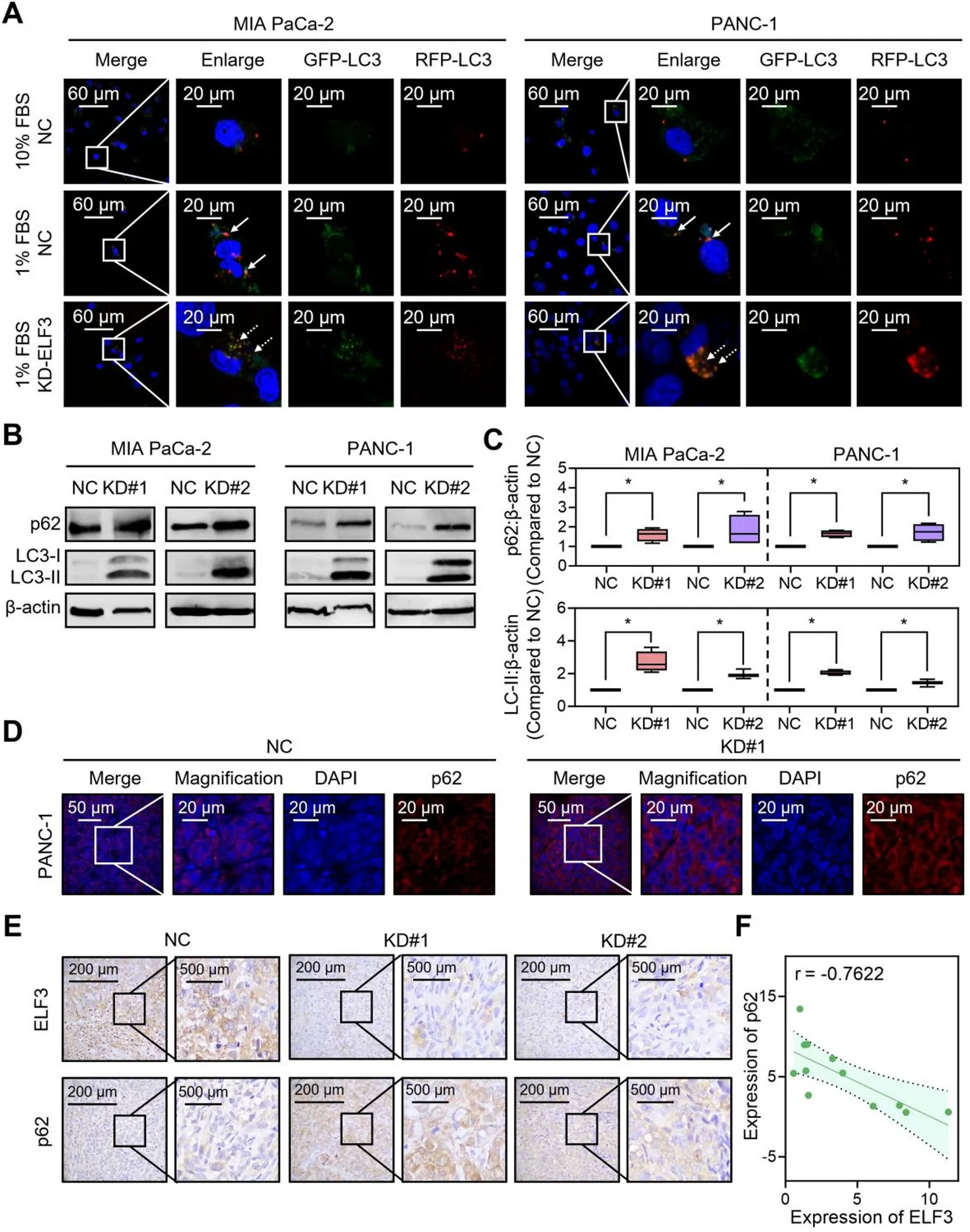
ELF3 knockdown blocks autophagic flux in vitro and in vivo. **A** GFP-RFP-LC3 showed that KD-ELF3 increased accumulation of autophagosomes (yellow puncta) and decreased the number of autolysosomes (red puncta). **B** and **C** Western blot indicated that KD-ELF3 significantly increased the level of p62 and LC3-II. **D** IF staining demonstrated that KD-ELF3 led to the accumulation of p62. **E** IHC staining confirmed that KD-ELF3 increased p62 in vivo. **F** Spearman correlation analysis revealed a significant negative correlation between ELF3 and p62 expression. *N* = 4 for (**C**, **E**); *N* = 6 for (**F**). *, *P* < 0.05

To investigate the relationship between ELF3 and autophagy, we first monitored autophagic flux using the RFP-GFP-LC3 reporter assay (Fig. 2A) and observed that under serum-starved conditions (1% fetal bovine serum [FBS]), cells displayed more autolysosomes (red puncta) than those maintained in complete medium (10% FBS). This confirmed that starvation induced autophagy. Notably, KD-ELF3 not only reduced autolysosome (red puncta) formation but also led to the accumulation of autophagosomes (yellow puncta), which suggests that KD-ELF3 impaired autophagic flux (Fig. 2A). This conclusion was further supported by immunoblot analysis which showed that KD-ELF3 significantly increased the levels of LC3-II and p62 (Fig. 2B and C), two established autophagic indicators that are normally degraded in autolysosomes. To further validate these observations in vivo, we performed IF and IHC staining and again observed that KD-ELF3 promoted the accumulation of p62 (Fig. 2D and E) in agreement with the western blot data. The spearman correlation supported the conclusion that ELF3 negatively regulated the level of p62 (Fig. 2F). These results suggested that KD-ELF3 blocked autophagy in vitro and in vivo.

**Fig. 3:**
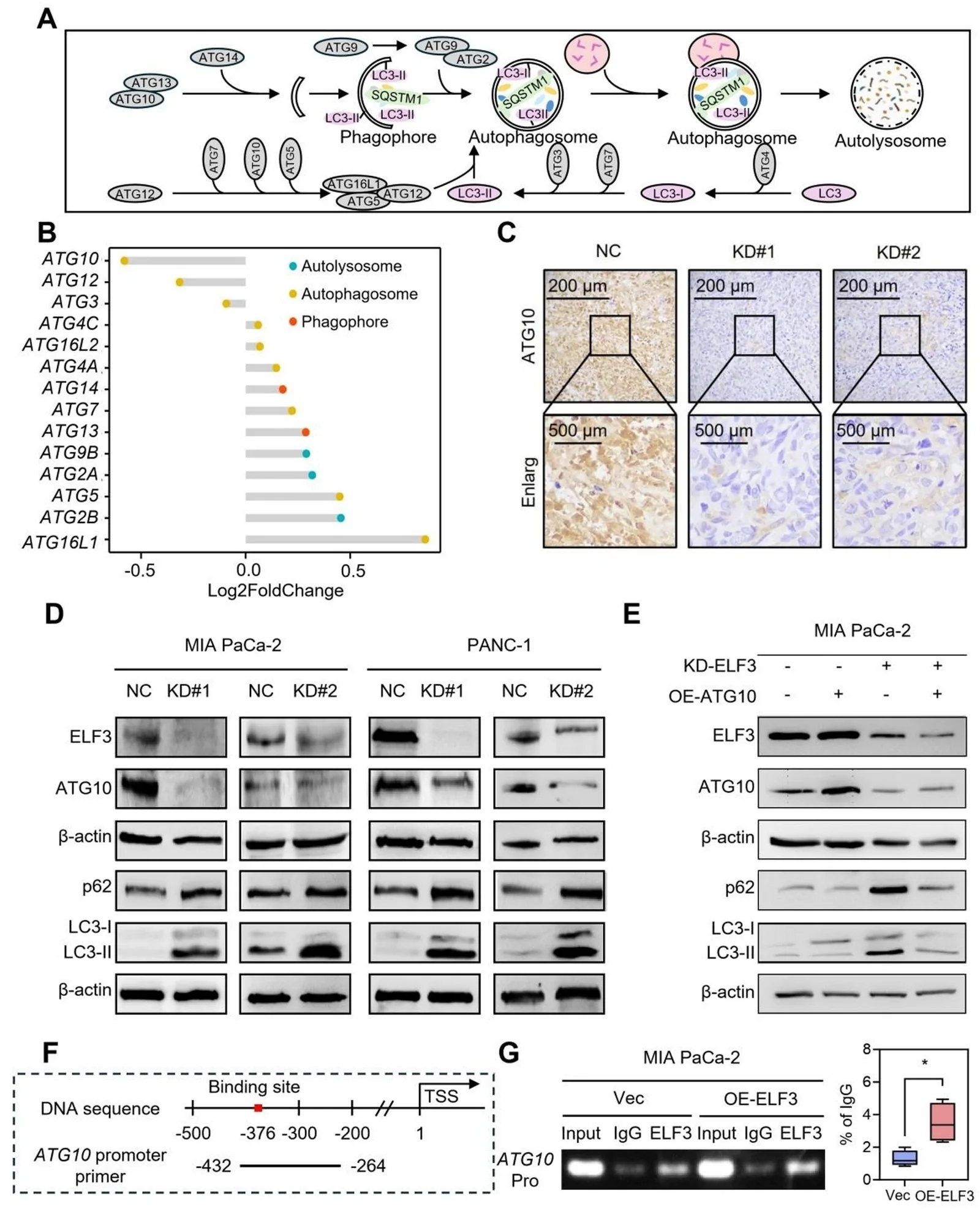
ELF3 promotes autophagy by transcriptionally activating *ATG10*. **A** Schematic illustration of the role of ATG proteins in autophagic flux. **B** The lollipop chart was used to visualize the expression of *ATG* genes after KD-ELF3. **C** IHC staining confirmed that KD-ELF3 decreased ATG10 in vivo. **D** Western blot indicated that KD-ELF3 decreased expression of ATG10 and increased the level of P62 and LC3-II. **E** Western blot analysis showed that OE-ATG10 restored autophagic flux which was impaired by KD-ELF3. **F** Schematic diagram of the *ATG10* promoter region and the primer for ChIP-PCR. **G** ChIP-PCR indicated that OE-ELF3 significantly enhanced the binding between ELF3 and the *ATG10* promoter. *N* = 4 for (**G**). *, *P* < 0.05

Autophagy is a pathophysiological process regulated by multiple ATG proteins (Fig. 3A). Thus, to elucidate the regulatory role of ELF3 in autophagy, we analyzed the expression of ATG genes (Fig. 3B) in ELF3-KO cells. It was observed that KD-ELF3 downregulated the expression of *ATG10* (Fig. 3B and C) and autophagic flux (Fig. 3D). To determine whether ATG10 mediates the regulation of autophagy by ELF3, rescue experiments were performed. We observed that overexpression of ATG10 (OE-ATG10) attenuated the KD-ELF3 mediated accumulation of p62 and LC3-II (Fig. 3E, lane 4 was compared to lane 3). This suggests that ATG10 acts as a downstream effector of ELF3 in the regulation of autophagy. Subsequently, ChIP-PCR was performed in ELF3-overexpressing cells (Fig. 3F and Fig. S2A and B), which proved that elevated overexpression of ELF3 (OE-ELF3) significantly enhanced ELF3 binding to the *ATG10* promoter (Fig. 3F and G). Collectively, these findings indicate that ELF3 functions as a transcriptional activator of *ATG10* by directly binding to the promoter of *ATG10*, thereby promoting autophagic flux.

### SRT2183 directly targets ELF3 and inhibits autophagy

**Fig. 4:**
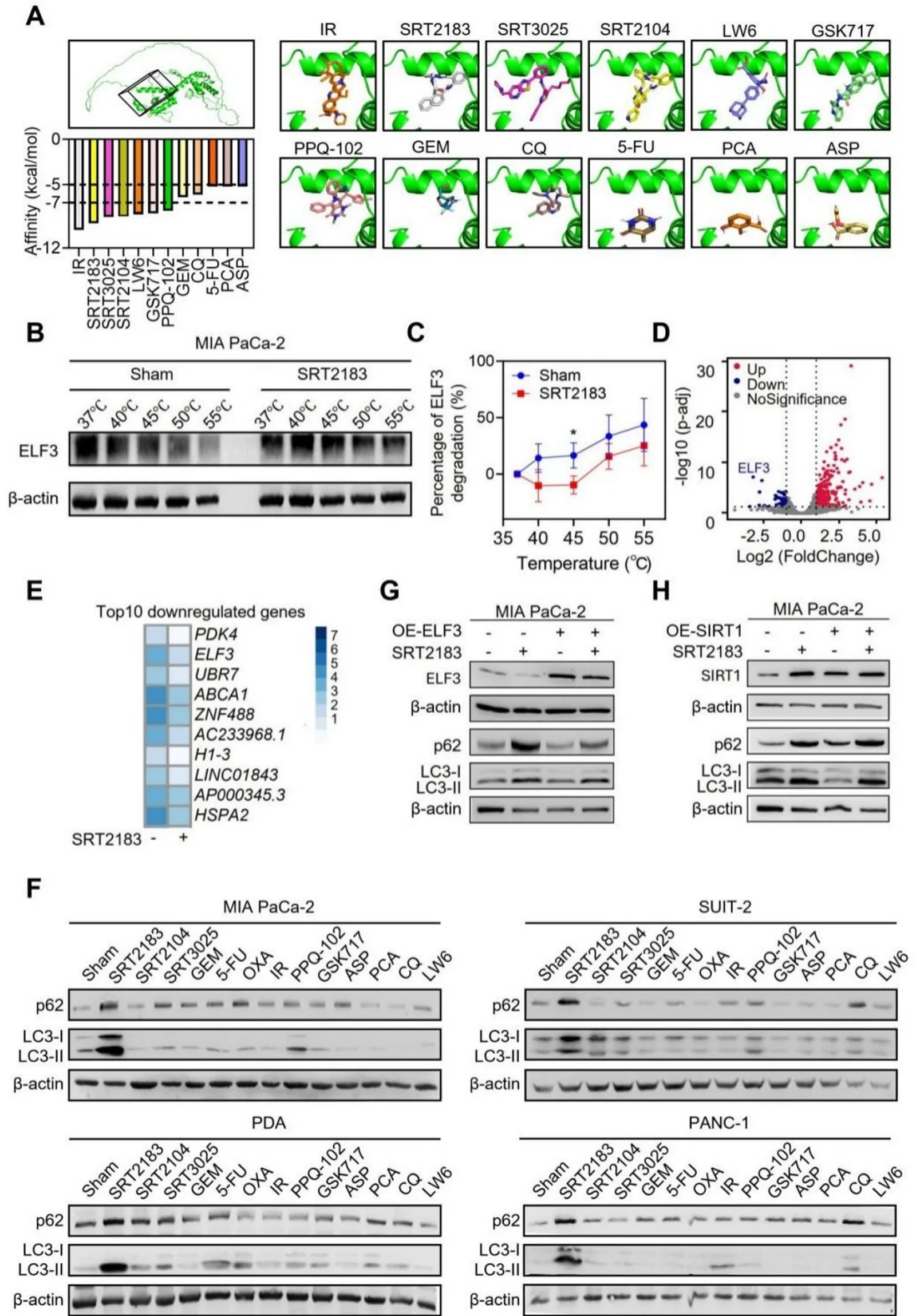
SRT2183 directly binds to ELF3 and disrupts ELF3-mediated autophagy flux. **A** Binding affinity analysis predicted strong interactions between ELF3 and IR, SRT2183, SRT3025 or SRT2104. **B** and **C** The cellular thermal shift assay showed that SRT2183 increased the thermal stability of ELF3 in MIA PaCa-2 cells compared with the Sham group. **D** Volcano plot showed differentially expressed genes between the Sham and SRT2183-treated groups. **E** ELF3 expression was significantly decreased following SRT2183 treatment. **G** Western blot analysis showed that OE- ELF3 reduced the SRT2183-mediated autophagy blockade (lane 2 was compared to lane 4). **H** Western blot analysis showed that OE-SIRT1 did not influent the accumulation of p62 and LC3-II induced by SRT2183 (lane 2 was compared to lane 4). **F** Western blot showed that SRT2183 significantly impaired autophagic flux in MIA PaCa-2, SUIT-2, PDA and PANC-1 cells, respectively. *N* = 4 for (**C**). *, *P* < 0.05

Currently, no inhibitors of ELF3 have yet been explored. We, therefore, evaluated the inhibitory effects of thirteen existing compounds in our laboratory (Table 1). Initial binding affinity calculations revealed that irinotecan (IR), SRT2183, SRT3025 and SRT2104 exhibited strong interactions with the ELF3 protein (Fig. 4A). Subsequently, to validate this finding, the cellular thermal shift assay was performed after the proteins were incubated with these compounds (Fig. 4B and Fig. S2C). The results demonstrated that SRT2183 significantly increased thermal stability of ELF3 (Fig. 4C). Additionally, mRNA sequencing proved that SRT2183 significantly decreased the level of *ELF3* (Fig. 4D and E). These results suggest that SRT2183 directly interacts with ELF3 and inhibits its expression. Moreover, the western blot proved that SRT2183 can inhibit autophagy (Fig. 4F). To evaluate whether the inhibitory effect of SRT2183 on autophagy is dependent on ELF3, ELF3-overexpressing MIA PaCa-2 cells were treated by SRT2183. We observed that OE-ELF3 attenuated SRT2183-mediated blockade autophagy (Fig. 4G, lane 2 was compared to lane 4). This indicated that ELF3 contributes to the inhibitory effect of SRT2183 on autophagic flux. Notably, SRT2183 was initially characterized as a Sirtuin 1 (SIRT1) activator [24]. To investigate if SIRT1 was also involved in SRT2183-mediated autophagy inhibition, we evaluated the expression of p62 and LC3-II in SIRT1-overexpressing cells, we observed that overexpression of SIRT1 (OE-SIRT1) did not influent the inhibition of autophagic flux by SRT2183 (Fig. 4H, lane 4 was compared to lane 2). These results demonstrated that ELF3 is a functional target of SRT2183 and plays an important role in SRT2183-mediated inhibition of autophagic flux.

### SRT2183 exhibits anti-carcinoma activity in vitro and in vivo

**Fig. 5:**
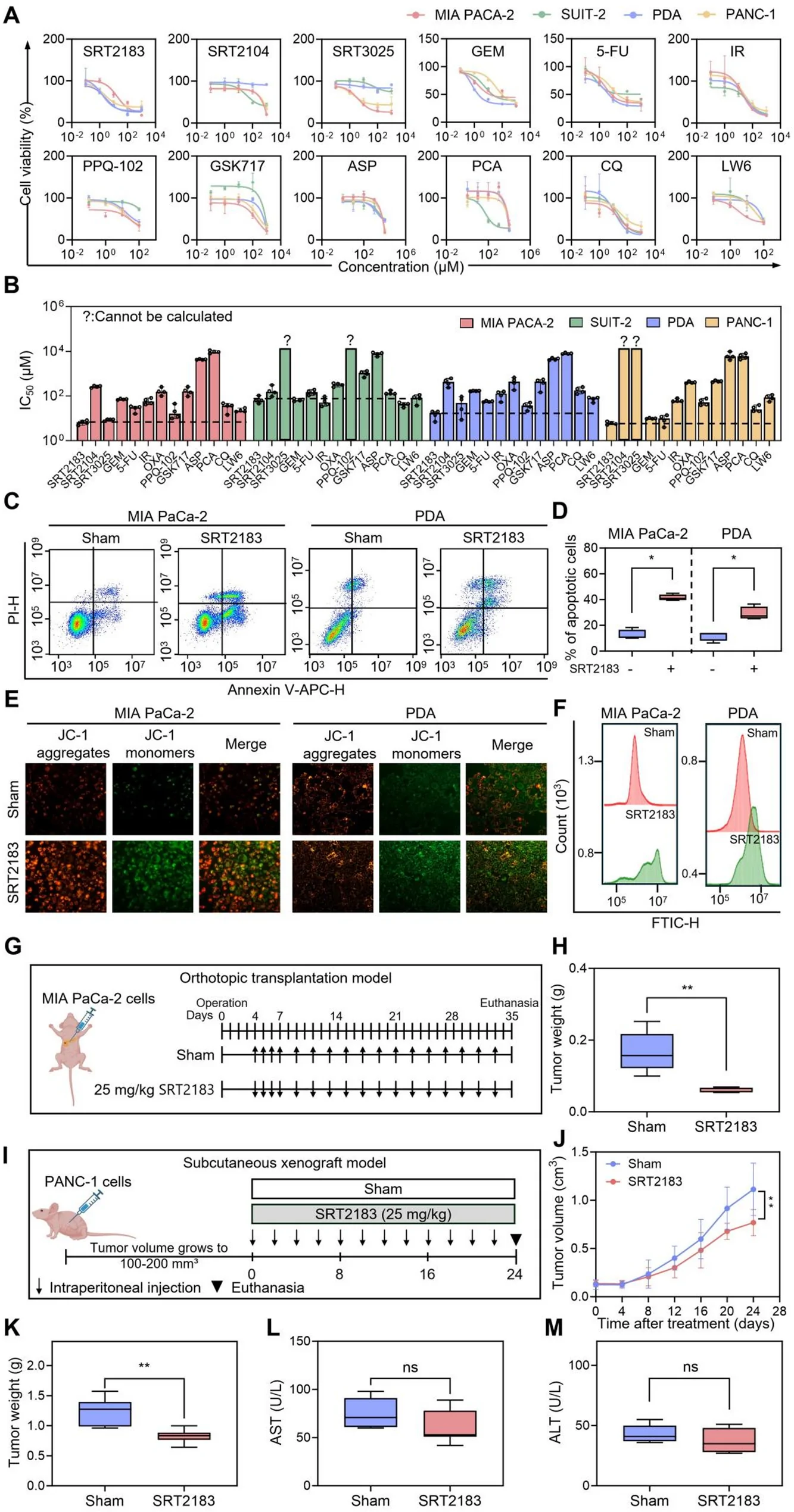
SRT2183 inhibits pancreatic tumor growth in vitro and in vivo. **A** CCK-8 assay indicated that SRT2183 significantly inhibited cell viability in MIA PaCa-2, SUIT-2, PDA and PANC-1 cells, respectively. **B** The IC₅₀ indicated that SRT2183 exhibited excellent anti-carcinoma activity in vitro. **C** and **D** Flow cytometry analysis demonstrated that SRT2183 significantly increased the percentage of apoptotic cells. **E** and **F** JC-1 staining assay confirmed that SRT2183 significantly induced cell death. **G** Schematic diagram of the orthotopic pancreatic cancer model. **H** Tumor weight was significantly reduced following SRT2183 treatment. **I** Schematic of the subcutaneous tumor model. **J** and **K** SRT2183 significantly reduced tumor volume and tumor weight in the subcutaneous tumor model. **L** and **M** AST and ALT levels indicated that SRT2183 did not induce hepatic toxicity. *N* = 4 for (**A**, **B**, **D**); *N* = 5 for (**H**); *N* = 7 for (**J**, **K**, **L**, **M**). *, *P* < 0.05; **, *P* < 0.01; ns, not significant

To evaluate the anti-carcinoma potential of SRT2183, cell viability was measured in the presence of different doses of SRT2183 and other compounds (Fig. 5A) and half-maximal inhibitory concentration (IC₅₀) were determined in MIA PaCa-2, SUIT-2, PDA and PANC-1 cells, respectively (Fig. 5B). We observed that SRT2183 exhibits excellent anti-carcinoma activity in all four cell lines (Fig. 5B). Additionally, the annexin V-propidium iodide analysis indicated that SRT2183 significantly induced cell apoptosis (Fig. 5C and D). This conclusion is supported by the JC-1 staining (Fig. 5E and F). To evaluate the anti-carcinoma activity of SRT2183in vivo, MIA PaCa-2 cells were orthotopically injected into the head of the pancreas in mice, which were subsequently intraperitoneally injected with 25 mg/kg of SRT2183 on day four after the operation (Fig. 5G). We observed that 25 mg/kg SRT2183 significantly decreased the tumor weight (Fig. 5H) and did not induce animal distress (Fig. S2E and F). To further assess the anti-carcinoma effect of SRT2183, MIA PaCa-2 cells were subcutaneously injected into mice and these mice were randomly treated with vehicle or 25 mg/kg SRT2183 every other day, when tumor volumes reached 100 to 200 mm^3^ (Fig. 5I). SRT2183 significantly decreased the tumor volume (Fig. 5J) and weight (Fig. 5K), while it did not increase the level of AST (Fig. 5L) and ALT (Fig. 5M).

## Discussion

The present study demonstrates for the first time that ELF3 promotes the transcription of *ATG10* and enhances the autophagic flux in PDAC. To elucidate this relationship between ELF3 and autophagy, we followed the guideline for the field and employed tandem RFP-GFP-LC3 fluorescence, western blot, IF and IHC analyses [11]. The results demonstrate that KD-ELF3 significantly impaired autophagic flux.

To clarify the mechanism of how ELF3 regulates autophagy, we analyzed the expression profiles of *ATG* genes, we found that KD-ELF3 differentially modulated the expression of *ATG* genes. For example, KD-ELF3 decreases the expression of *ATG10*, *ATG12* and *ATG3*, while it increases the expression of other ATG genes (Fig. 3B). This could be explained by the hypothesis that KD-ELF3 inhibits the transcription of *ATG10* and blocks the initiation of autophagy (Fig. 3A). As the blocking time increases, it is likely to induce a secondary autophagic response triggered by the accumulated undigested autophagic substrates [11]. This secondary autophagic response may influence the expression of the other ATG genes. However, this should be investigated by further studies.

Although the current study demonstrates that KD-ELF3 significantly suppresses cell proliferation and tumor growth in PDAC, the generalizability of these findings, as well as the potential therapeutic benefits of targeting ELF3, in other cancer types or distinct pancreatic cancer subtypes remains to be further investigated. With the support of Gene Expression Profiling Interactive Analysis [25], we systematically analyzed ELF3 expression patterns across 31 cancer types in a pan-cancer analysis, and observed that compared to the normal tissues ELF3 was significantly highly expressed in cervical squamous cell carcinoma and endocervical adenocarcinoma, cholangiocarcinoma, colon adenocarcinoma, kidney renal papillary cell carcinoma, lung adenocarcinoma, ovarian serous cystadenocarcinoma, pancreatic adenocarcinoma, rectum adenocarcinoma, stomach adenocarcinoma, testicular germ cell tumors, thyroid carcinoma, thymoma, uterine corpus endometrial carcinoma and uterine carcinosarcoma (Fig. S3A). Conversely, ELF3 displayed significantly decreased expression in head and neck squamous cell carcinoma, kidney chromophobe, sarcoma and skin cutaneous melanoma (Fig. S3A). Additionally, the Kaplan-Meier analysis demonstrated that targeting ELF3 may only benefit patients with PDAC (Fig. 1B) or ovarian serous cystadenocarcinoma (Fig. S3B). Moreover, inhibiting the expression of ELF3 may promote the tumor growth in ampullary carcinoma [26, 27]. For example, Yachida et al. reported that silencing ELF3 enhances motility and invasion of epithelial cells [26]. These results demonstrated that the oncogenic function of ELF3 is likely context-dependent; therefore, ELF3-targeted anti-tumor therapies require individualized selection based on specific tumor types.

SRT2183 is a small-molecule compound that was initially reported to bind to an allosteric site of SIRT1 [24]. This interaction was proposed to induce conformational changes in SIRT1, thereby enhancing its affinity for specific substrates involved in cellular metabolism, aging, and stress responses. However, subsequent studies suggested that the biological effects of SRT2183 may not be mediated through SIRT1 activation [28, 29]. Consistent with these findings, our results indicate that the inhibitory effect of SRT2183 on autophagy is not dependent on SIRT1 activation (Fig. 4H). Further investigations identified ELF3 as a functional target of SRT2183 and demonstrated that targeting ELF3 contributes to SRT2183-mediated suppression of autophagic flux and pancreatic cancer growth (Fig. 4G).

CQ is a U.S. Food and Drug Administration approved drug for the treatment of malaria, rheumatoid arthritis, and systemic lupus erythematosus [30, 31]. Recently CQ has emerged as the most widely used autophagy inhibitor in cancer research and is being evaluated in clinical trials (e.g., NCT01777477). Thus, we also evaluated the benefits of SRT2183 in combination with CQ for fighting against pancreatic cancer. The CCK-8 analysis demonstrated that SRT2183 plus CQ synergistically inhibited cell viability when compared to each monotherapy (Fig. S4A and B). The western blot demonstrated that SRT2183 significantly inhibited the expression of ATG10 (Fig. 4G), which is necessary for the initiation of autophagy. Additionally, it is well documented that CQ inhibits autophagy by impairing autophagosome fusion with lysosomes [32]. Thus, the synergistic effect of SRT2183 and CQ may be dependent on blockade of different autophagy stages by these two compounds. This conclusion is supported by western blot, which demonstrate that the combination therapy achieves superior inhibitory activity of autophagy compared to monotherapy (Fig. S4C and D).

Although our findings demonstrate that KD-ELF3 suppresses cell proliferation and tumor growth in pancreatic cancer, it has been reported that ELF3 exerts context-dependent functions, acting as either an oncogenic or tumor-suppressive factor depending on the cellular environment and molecular background [16]. Therefore, further studies are required to determine the therapeutic applicability of ELF3 inhibition and SRT2183 in other malignancies and to identify cancer types that may benefit most from ELF3-targeted therapies.

Furthermore, the present study demonstrates that SRT2183 suppresses ELF3-mediated autophagic flux and inhibits tumor growth. However, RNA sequencing analysis revealed that SRT2183 significantly modulated some signaling pathways, including oxytocin, p53, and Hedgehog signaling pathways (Fig. S4E and F). These findings suggest that SRT2183 may exert its anti-carcinoma effects through distinct molecular mechanisms in addition to ELF3-mediated regulation of autophagy. Moreover, dysregulation of these signaling pathways may be associated with potential organ toxicity. For example, aberrant activation of the p53 pathway has been implicated in apoptosis and nephrotoxic acute kidney injury [33]. In addition, abnormal Hedgehog signaling has been reported to contribute to renal fibrosis through the activation of fibroblasts and promotion of extracellular matrix deposition [34]. Nevertheless, the present study did not specifically evaluate the potential nephrotoxicity associated with SRT2183 treatment. Therefore, future studies are warranted to specifically investigate the potential nephrotoxicity of SRT2183 through comprehensive evaluation of renal function, renal histopathological changes, and molecular markers associated with kidney injury in appropriate preclinical models. Such investigations will be essential for determining the renal safety profile and further evaluating the clinical feasibility of SRT2183.

Last but not least, we acknowledge that the selection of the ELF3 inhibitor in the present study was not based on extensive compound screening. Therefore, future studies should leverage artificial intelligence-based approaches and large-scale chemical and biological databases to systematically identify, optimize, and validate more potent and selective ELF3-targeting inhibitors.

## Conclusion

In conclusion, this study demonstrates that ELF3 promotes pancreatic cancer progression and identifies ELF3 as a potential therapeutic target. Mechanistically, it reveals that ELF3 promotes autophagy through transcriptional upregulation of ATG10, thereby contributing to pancreatic cancer development. SRT2183, an ELF3-targeting compound, suppresses ELF3-mediated autophagic regulation and results in inhibition of tumor growth.

## Supplementary Information

Supplementary Material 1.

## Data Availability

All the data and materials are available on reasonable request from corresponding author.

## Abbreviations

ALT: Alanine aminotransferase
AST: Aspartate aminotransferase
5-FU: 5-fluorouracil
ATG: Autophagy-related
CESC: Cervical squamous cell carcinoma and endocervical adenocarcinoma
CETSA: Cellular thermal shift assay
ChIP: Chromatin immunoprecipitation
CHOL: Cholangiocarcinoma
CLDN7: Claudin 7
COAD: Colon adenocarcinoma
CQ: Chloroquine
EdU: 5-ethynyl-2'-deoxyuridine
ELF3: E74 like transcription factor 3
KD-ELF3: Knockdown of ELF3
OE-ELF3: Overexpression of ELF3
ETS: E-twenty-six
FBS: Fetal bovine serum
GEM: Gemcitabine
GEO: Gene Expression Omnibus
GOT1: Glutamic-oxaloacetic transaminase 1
GPT: Glutamic-pyruvic transaminase
GTEx: Genotype-Tissue Expression
HNSC: Head and neck squamous cell carcinoma
IC₅₀: Half-maximal inhibitory concentration
IHC: Immunohistochemistry
KICH: Kidney chromophobe
KIRP: Kidney renal papillary cell carcinoma
LUAD: Lung adenocarcinoma
NC: Negative control
OV: Ovarian serous cystadenocarcinoma
PAAD: Pancreatic adenocarcinoma
PCR: Polymerase chain reaction
PDAC: Pancreatic ductal adenocarcinoma
PLIP: Protein-ligand interaction profiler
qRT-PCR: Quantitative reverse transcription PCR
READ: Rectum adenocarcinoma
SARC: Sarcoma
shRNAs: Short hairpin RNAs
SIRT1: Sirtuin 1
SKCM: Skin cutaneous melanoma
STAD: Stomach adenocarcinoma
TCGA: The Cancer Genome Atlas
TGCT: Testicular germ cell tumors
THCA: Thyroid carcinoma
THYM: Thymoma
UCEC: Uterine corpus endometrial carcinoma
UCS: Uterine carcinosarcoma
