# Supplementary material for: ELF3 (E74 like ETS transcription factor 3) promotes tumor growth and autophagy in pancreatic cancer
Source: J Exp Clin Cancer Res. 2026 Aug 18;45:236. doi: 10.1186/s13046-026-03806-3 (PMC13757040; doi:10.1186/s13046-026-03806-3)
Supplement: Supplementary file 1 [file 13046_2026_3806_MOESM1_ESM.docx]

**Figure S1.** High ELF3 expression serves as an independent risk factor for poor prognosis in PDAC. **(A)** Flowchart of inclusion and exclusion criteria for the TCGA-PAAD cohort. **(B)** Kaplan-Meier analysis indicated that high *ELF3* expression was associated with poor prognosis in PDAC patients using the GEO GSE85916. **(C)** Forest plot depicting the multivariate Cox proportional hazards regression analysis, showing that high *ELF3* expression is an independent predictor of poor overall survival in patients with PDAC (HR = 1.74, 95% CI: 1.053–2.9, P = 0.031).

**Figure S2. Analysis of ELF3 binding to the ATG10 promoter, candidate compound interactions with ELF3, and SRT2183 tolerability in mice. (A)** Schematic diagram of the *ATG10* promoter used for ChIP-PCR analysis. **(B)** ChIP-PCR indicated that overexpression of ELF3 (OE-ELF3) did not significantly enhance its binding to the ATG10 promoter at Pro2, Pro3, or Pro4 regions. **(C)** Cellular thermal shift assay demonstrated that IR, SRT3025 and SRT2104 did not increase the stability of ELF3 and indicated that these compounds did not directly interact with ELF3 in cells. **(D)** Schematic diagram illustrated the orthotopic pancreatic cancer model and drug administration protocol. **(E)** The animal distress score indicated that daily injections of 100 mg/kg or 50 mg/kg SRT2183 resulted in mortality or severe distress in mice. N = 4 for (C); N = 3 for (F). ns, not significant.

**Figure S3.** *ELF3* expression across different tumor types and its association with patient survival. **(A)** Box plots revealed that *ELF3* was significantly highly expressed in CESC, CHOL, COAD, KIRP, LUAD, OV, PAAD, READ, STAD, TGCT, THCA, THYM, UCEC and UCS. Conversely, *ELF3* was significantly expressed at a lower level in HNSC, KICH, SARC and SKCM. **(B)** Kaplan–Meier survival curves and log-rank test demonstrated that high *ELF3* expression was significantly associated with poor survival in patients with OV; however, it did not influent the survival of patients with other cancers. *, P < 0.05.

**Figure S4.** SRT2183 exerts multi-target effects and enhances the efficacy of chloroquine (CQ) in PDAC cells. **(A and B)** SRT2183 increased the anti-carcinoma activity of CQ. **(C and D)** Western blot analysis showed that compared to CQ, SRT2183 in combination with CQ increased the level of LC3-II and p62. **(E)** Bulk RNA sequencing suggested that in addition to regulating autophagy in pancreatic tumor cells, SRT2183 is also involved in modulating apoptosis, metastasis, and proliferation. **(F)** GSEA enrichment analysis plot indicated that SRT2183 regulated some signaling pathways, such as oxytocin, p53, and Hedgehog signaling pathways. N = 4 for (A, B). *, P < 0.05.

**Supplementary Table**

| **Table S1. shRNA sequences used for ELF3 knockdown** | | |
| --- | --- | --- |
| shRNA Name | Sequence (5’→3’) | Target |
| ELF3 Control | TTCTCCGAACGTGTCACGT | Negative control |
| ELF3 KD#1 | gcCATGAGGTACTACTACAAA | *ELF3* |
| ELF3 KD#2 | gcTCTTCTGATGAGCTCAGTT | *ELF3* |
